# The PRECious trial PREdiction of Complications, a step-up approach, CRP first followed by CT-scan imaging to ensure quality control after major abdominal surgery: study protocol for a stepped-wedge trial

**DOI:** 10.1186/s13063-015-0903-y

**Published:** 2015-08-28

**Authors:** Jennifer Straatman, Miguel A. Cuesta, W. H. (Hermien) Schreurs, Boudewijn J. Dwars, Huib A. Cense, Herman Rijna, D. J. A. (Eric) Sonneveld, Frank C. den Boer, Elly S. M. de Lange- de Klerk, Donald L. van der Peet

**Affiliations:** Department of Gastrointestinal Surgery, VU Medical Center, De Boelelaan 1117, 1081, HV Amsterdam, The Netherlands; Department of Surgery, Medisch Centrum Alkmaar, Wilhelminalaan 12, 1815, JD Alkmaar, The Netherlands; Department of Surgery, Slotervaart Ziekenhuis, Louseweg 6, 1066, EC Amsterdam, The Netherlands; Department of Surgery, Rode Kruis Ziekenhuis, Vondellaan 13, 1942, LE Beverwijk, The Netherlands; Department of Surgery, Spaarne Gasthuis, Vondelweg 999, 2026, BW Haarlem, The Netherlands; Department of Surgery, Westfries Gasthuis, Maelsonstraat 3, 1624, NP Hoorn, The Netherlands; Department of Surgery, Zaans Medisch Centrum, Koningin Julianaplein 58, 1502, DV Zaandam, The Netherlands; Department of Epidemiology and Biostatistics, VU Medical Center, De Boelelaan 1117, 1081, HV Amsterdam, The Netherlands

**Keywords:** Major abdominal surgery, postoperative complications, quality control, C-reactive protein, computed tomography

## Abstract

**Background:**

After major abdominal surgery (MAS), 20 % of patients endure major complications, which require invasive treatment and are associated with increased morbidity and mortality. A quality control algorithm after major abdominal surgery aimed at early identification of patients at risk of developing major complications can decrease associated morbidity and mortality. Literature studies show promising results for C-reactive protein (CRP) as an early marker for postoperative complications, however clinical significance has yet to be determined.

**Methods:**

A multicenter, stepped wedge, prospective clinical trial including all adult patients planned to undergo elective MAS. The first period consists of standard postoperative monitoring, which entails on demand additional examinations. This is followed by a period with implementation of postoperative control according to the PRECious protocol, which implicates standardized measurement of CRP levels. If CRP levels exceed 140 mg/L on postoperative day 3,4 or 5, an enhanced CT-scan is performed.

Primary outcome in this study is a combined primary outcome, entailing all morbidity and mortality due to postoperative complications. Complications are graded according to the Clavien-Dindo classification. Secondary outcomes are hospital length of stay, patients reported outcome measures (PROMs) and cost-effectiveness. Data will be collected during admission, three months and one year postoperatively. Approval by the medical ethics committee of the VU University Medical Center was obtained (ID 2015.114).

**Discussion:**

the PRECious trial is a stepped-wedge, multicenter, open label, prospective clinical trial to determine the effect of a standardized postoperative quality control algorithm on postoperative morbidity and mortality, and cost-effectiveness.

**Trial registration:**

www.ClinicalTrials.gov, NCT02102217. Registered 5 February 2015.

**Electronic supplementary material:**

The online version of this article (doi:10.1186/s13063-015-0903-y) contains supplementary material, which is available to authorized users.

## Background

Major abdominal surgery (MAS) may be defined as a surgical resections performed on colorectal, hepato-pancreatico-biliary and upper gastrointestinal organs with either primary anastomosis and/or stoma. Around 20 % of all MAS patients have a major complication, which requires invasive treatment such as reoperation, percutaneous drainage and intensive care admission [1, 2]. Major complications further increase morbidity and mortality after MAS, leading to longer intensive care and hospital stay, unplanned open procedures, creation of stomas and possible increase in cancer recurrence rates and costs [3–5].

In current postoperative practice median time to clinical diagnosis of a postoperative complication is approximately 8 days [6–8]. A delay in diagnosis of complications increases morbidity and mortality related to major complications [9–11], whereas early diagnosis of postoperative septic complications, before clinical deterioration, is associated with a decrease in the associated morbidity and mortality [12, 13]. Early detection of septic complications is challenging and may (clinically, serologically and in imaging techniques) be hard to distinguish from the physiological and postoperative systemic inflammatory response syndrome (SIRS) [8, 14, 15]. Furthermore clinical risk assessment appears to have a low predictive value for major complications such as anastomotic leak [16]. This further stresses the need for a standardized quality control algorithm after MAS. Notably, there are currently no standard quality control tests or protocols available for use after MAS to differentiate between a normal and a complicated postoperative course.

Several biomarkers, such as Interleukin-6 (IL-6), tumor necrosis factor α (TNF-α), procalcitonin, white blood cell count (WBC) and C-reactive protein (CRP) [17], have been assessed in the search for a marker in the early diagnosis of postoperative complications. WBC does not significantly differ in patients with an uncomplicated versus complicated postoperative course. WBC is therefore not useful in a standardized quality control algorithm. Procalcitonin, IL-6 and TNF-α have been assessed as markers of postoperative sepsis [18]. However, compared to CRP they achieve similar results in predicting major complications after MAS. Moreover, taking into account the higher cost and limited availability of procalcitonin, IL-6 and TNF-α testing [19], further focus will be on the use of CRP in a postoperative standardized quality control algorithm.

CRP is well-established as a marker of infections and complications and has shown promising results [19, 20]. It is an acute-phase protein synthesized in the liver under stimulation of IL-6 and TNF-α in inflammatory processes, which amongst others enhances phagocytosis of bacteria by macrophages [21]. In healthy individuals, the CRP level is <1 mg/L. In mild inflammation it can rapidly reach over 40 mg/L and even levels >400 mg/L have been detected in severe inflammatory response, sepsis or burns [22]. CRP levels also increase as a consequence of operative trauma, and it is even suggested that the level of postoperative CRP is proportional to the length of the operation, amount of operative trauma and intra-operative complications [23]. A peak in postoperative CRP levels is observed 48 − 72 hours after surgery. In uneventful cases, the levels decrease after this peak [24]. Furthermore, the plasma half-life of CRP is 19 hours and postoperative CRP levels are independent of gender, age, body mass index (BMI), diet, diurnal rhythm or organ function [25]. Circulating CRP levels are therefore only determined by their rate of synthesis [26]. Based on these characteristics, CRP might be a promising valuable marker for grading inflammation related to postoperative complications.

Several studies have assessed the use of CRP as a marker for postoperative complications after MAS. Established cut-off CRP levels serving as markers for infective complications range from 140 mg/L to 170 mg/L on postoperative day (POD) 3 [27, 28]. In relation to anastomotic leakage in patients undergoing colorectal surgery, a cutoff for CRP of 190 mg/L on POD 3 and 125 mg/L on POD 4 has been proposed [29, 30]. The largest retrospective series included 1,187 patients who had undergone colorectal surgery, calculating a cutoff of 123 mg/L as a marker for all septic complications [20, 31], yet they did not differentiate between minor and major complications. A recent meta-analysis established a cutoff of 172 mg/L on POD 3 as a marker for anastomotic leakage [32]. Definitions for anastomotic leak vary widely in the literature [33], which limits reproducibility and excludes patients that require re-intervention for other complications. Therefore, our interest lies in diagnosing all major complications that require invasive treatment, as classified by grades 3 − 5 in the Clavien-Dindo classification [34, 35].

Based on our own retrospective data for 399 patients who underwent MAS, a level of 140 mg/L is proposed as cutoff for POD 3, 4 and 5 as a marker for major complications, with an overall sensitivity of 78.1 %, a specificity of 53.7 % and a negative predictive value of 89.1 %. This is in line with recent literature [2, 36].

Serum CRP is non-specific for location, thus, additional imaging is required. Computed tomography (CT) is the current imaging modality of choice. In our retrospective data, CT had sensitivity of 91.7 % and specificity of 100 % for diagnosis of major complications; this is confirmed in the literature [14, 37]. Moreover, in the study conducted by Eckmann et al. CT had sensitivity of 97 % [13]. Another recent study established CT as the preferred modality in diagnosis of anastomotic leakage [36].

In 2008, Den Dulk et al. implemented a standardized scoring system for the clinical status of patients undergoing colorectal surgery. With this system they decreased the time between surgery and diagnosis of anastomotic leakage from 8 to 6 days, thereby decreasing mortality from 39 % to 24 % (*p* = 0,24), further supporting the role of a standardized postoperative quality control algorithm following MAS [6, 7]. However the search for an optimal algorithm continues. CRP and CT scanning have been shown to differentiate between an uncomplicated and a complicated postoperative course. Currently their use is only on demand. The PRECious protocol presented here is a postoperative quality control algorithm, which is aimed at early diagnosis and treatment of patients with major complications.

## Methods

### Study objectives

The aim of this study is to evaluate the role of a standardized quality control algorithm after MAS in order to early diagnose and treat major complications and achieve safe discharge criteria. The PRECious trial is a prospective, multicenter, open, stepped-wedge study. All patients who are planned to undergo elective major digestive surgery, defined as any gastro-intestinal resection with reconstruction via anastomosis or stoma, will be included. Our hypothesis is that the standardized postoperative quality control algorithm will allow for early diagnosis and treatment of complications, thereby decreasing morbidity and mortality associated with MAS.

### Endpoints

The primary endpoint of the study consists of a combined endpoint entailing all morbidity and mortality due to major complications. Complications will be classified according to a modified Clavien-Dindo classification in two groups [34, 35]. Group I consists of grade I and grade II complications, which are classified as minor. Early detection of major complications that may require invasive treatment, such as re-laparotomy or percutaneous drainage and/or intensive care admission, which might even lead to death, as classified by Clavien-Dindo grades III, IV and V, is of major importance. The comprehensive complication index will also be calculated [38]. All mortality and morbidity after major complications will be recorded, entailing fistula; bowel obstruction or herniation; abscesses; wound dehiscence; abdominal compartment syndrome; unplanned enterostomy; enterostomy dysfunction; myocardial infarction; pulmonary embolism; respiratory insufficiency; urosepsis; cerebrovascular accident; bleeding, and anastomotic leak.

Secondary endpoints will be patient-related outcome measures (PROMs), measured with the EuroQol-5D (EQ-5D) and gastrointestinal quality of life index (GIQLI) questionnaires. Furthermore postoperative recovery data will be collected in both groups, such as length of hospital stay and intensive care length of stay. Alongside the trial a cost-effectiveness analysis will be conducted.

### Power of the study

Based on retrospective data of 399 patients who underwent MAS, we assessed all mortality and morbidity due to major complications (multiple reoperations, multiple percutaneous drainages, enterostomy during re-intervention) and found this to be 17 %. Based on the results of den Dulk, who report a decrease in mortality following implementation of a clinical item scoring list, we calculated the following sample size [6]: a group sample size of 525 in group 1 and 525 in group 2 to achieve 80 % power to detect a difference in frequency of morbidity in the control group, which is 17 %, in comparison to the PRECious group, with an expected morbidity of 11 %. The test statistic used is the two-sided Mantel-Haenszel test. The significance level of the test was targeted at 0.05.

### Inclusion criteria

All adult patients who are to undergo elective MAS can be included in the study. MAS will be defined as all gastrointestinal resections with reconstruction with anastomosis and/or stoma. For instance, cholecystectomy will not be included because no anastomosis or stoma is performed. Written informed consent will be obligatory.

### Exclusion criteria

Patients who undergo emergency surgery as a primary operation will be excluded to allow for appropriate informed consent. Patients with an American Society of Anaesthesiologists (ASA) classification of four of higher will be excluded. As CT may be performed if CRP levels exceed 140 mg/L, patients with pre-operative impaired renal function (glomerular filtration rate <60) (within 4 weeks before surgery) will be excluded. Also patients with allergy to contrast medium will be excluded.

### Participating surgeons and clinics

Seven Dutch hospitals will participate in the study; one academic and six teaching centers: VU medical center, Amsterdam; Slotervaart ziekenhuis, Amsterdam; Medisch Centrum Alkmaar, Alkmaar; Rode Kruis Ziekenhuis, Beverwijk; Spaarne Gasthuis, Haarlem; Westfries Gasthuis, Hoorn and Zaans Medisch Centrum, Zaandam. All participating surgeons have ample experience within their respective field. This is of importance because complication rates and morbidity are associated with learning curves and might therefore affect the primary outcome [39].

### Patient allocation and design

Patients will be informed about the study at the outpatient clinic. The patient will be included upon admission after informed consent has been obtained. Due to the nature of the study, a parallel-randomized design is not the preferred option, because this would allow for bias by crossover and automated CRP and CT enquiries by physicians in both patient groups. A cluster design is not deemed feasible due to differences between the participating centers (i.e., academic and teaching hospitals). A stepped-wedge design is deemed appropriate. Due to the large number of participating centers, little bias is expected from other changes in treatment. Changes will be monitored and corrected for if applicable. Thus, the two groups will be compared according to two periods. Patients allocated to the first period will receive standard on-demand additional examinations. After a transition phase of one month allowing for implementation, all patients will be allocated to the intervention arm and will receive standardized postoperative monitoring of serum CRP levels on POD 3, 4 and 5. If CRP levels exceed 140 mg/L additional CT will be conducted.

Data collection will be via an online module, which allows for inclusion and data collection. Participating surgeons will be able to log in to the secured module via the PRECious trial website. After completing the inclusion form, an immediate response with a code number and type of postoperative control protocol will be obtained.

The nature of the study does not allow for blinding, because it would not be ethical to perform sham venous puncture, and the protocol will be known by the attending physician to allow CT to be arranged. Instead of blinding, the add-on value of CRP measurements will be tested. The attending physician will have to grade the patient in the morning before CRP levels are known, grade 1 indicating a healthy patient, grade 10 a patient at acute risk of death. The physician will also have to state whether he/she would perform CT prior to CRP levels being measured, allowing for assessment of the add-on value of the presented standardized quality control algorithm.

### Data collection and statistics

Data will be collected partially by means of a secured Internet module and partially by hard-copy datasheets. The secured online module is especially designed for the PRECious trial. Hard-copy datasheets, such as completed quality of life questionnaires will be sent to the VU medical center by mail, were they will be kept in a secured room. Data will be collected daily until discharge. PROMs will be collected preoperatively, and at 5 days, 3 months and 12 months postoperatively. A study chart for measurements is depicted in Table 1.

**Table 1:** Study measurements

	Study period										
	Enrolment	Allocation	Post-allocation								Close-out
Time point	Pre-operative	Pre-operative	Surgery	POD 2	POD 3	POD 4	POD 5	Discharge	3 Mo	12 Mo	t_x_
Enrolment											
Eligibility screen	X										
Informed consent	X										
Allocation		X									
Interventions											
Control group		X									
CRP measurement				X	X	X	X				
QOL questionnaire		X					X		X	X	
PRECious group											
CRP measurement				X	X	X	X				
CT if CRP >140 mg/L					X	X	X				
QOL questionnaire		X					X		X		
Assessments											
Control group											
List baseline variables		X									
List operative data			X								
List admission data								X			
Quality of life		X					X		X	X	
List morbidity								X	X	X	
List survival								X	X	X	
PRECious group											
List baseline variables		X									
List operative data			X								
List admission data								X			
Quality of life		X					X		X	X	
List morbidity								X	X	X	
List survival								X	X	X	X

*POD* postoperative day, *Mo* months, *CRP* C-reactive protein, *CT* computed tomography scan, *QOL* Quality of Life, T_x_ end of protocol

One research fellow in the VU medical center will monitor the data for all included patients, and maintain regular contact with all participating centers. Due to the nature of the study the researcher cannot be blinded, hence an external monitoring committee (Clinical Research Bureau, Amsterdam, Netherlands) is installed and will assess data collection. All required parameters will be collected in an SPSS data file. Data analysis will be performed according to the intention-to-treat principle. Continuous variables will be compared using the *t* test or Mann-Whitney *U* test, as appropriate. Frequencies will be compared using the Chi-square or McNemar tests, as appropriate.

### Economic evaluation

Direct medical costs, non-medical costs and time-cost differences will be calculated for each arm of the study. These will include the increased costs in the PRECious arm due to the extra CRP testing and greater use of CT, and costs due to complications and readmissions. We expect admission duration to be shorter in the PRECious arm due to early diagnosis and treatment of complications. Therefore, the protocol committee deems the protocol cost-effective. On retrospective analysis of direct medical costs in patients who underwent MAS the average cost was 8.584,81 € (95 % CI 8.332,51 − 8.860,81 €) in patients without complications, 15.412,96 € (95 % CI 14.250,22 − 16.708,82 €) after minor complications, and 29.198,23 € (95 % CI 27.187,13 − 31.295, 78 €) in patients with a major complication that required invasive treatment (*p* <0.001) [5].

### Ethics

The study will be conducted in accordance with the principles of the Declaration of Helsinki and Good Clinical Practice guidelines. The protocol has been written in concordance with the SPIRIT guidelines, as depicted in the checklist (see Additional file 1) [40]. The scientific research committee of the Cancer Center Amsterdam, the Netherlands, approved the study on 12 March 2014 (ID: Pro 14/23). Approval by the medical ethics committee of the VU University Medical Center, Amsterdam was obtained on 26 May 2015 (Protocol ID 2015.114 – NL43534.029.15). The board of executives in each participating center will have to approve the final protocol (version 2.0, 4 May 2015) prior to the start of inclusions. Written informed consent will be obtained from all participating patients. This trial was registered at clinicaltrials.gov on 5 February 2015, with trial number NCT02102217.

### Postoperative quality control

#### Preoperative preparation

Patients in both groups will receive similar preoperative treatment. Standard anti-thrombotic prophylaxis will be administered according to the local protocol. Furthermore, standard prophylactic antibiotics will be administered according to the local protocol.

#### Standard group

In the first period all patients will be allocated to the control group and will receive standard postoperative monitoring, which consists of daily measurement of clinical parameters (heart rate, blood pressure, temperature, pain) and assessment of the patient by the attending physician. Additional examinations such as blood sampling and imaging will only be conducted on demand, for instance, if clinical parameters deviate or physical examination shows signs of complications.

#### PRECious group

The second period will be considered the intervention group. All patients will be allocated to postoperative controls according to the PRECious protocol. Within this protocol peripheral blood samples for CRP measurements will be collected routinely on POD 2, 3, 4 and 5. If CRP exceeds 140 mg/L on the third, fourth or fifth POD, enhanced CT of the abdomen will be conducted within 24 hours. It is expected that two thirds of patients will undergo CT versus one third of the control group. Intravenous contrast is administered, with regard to renal function, to conduct CT. Depending on the organ undergoing surgery, oral or rectal contrast will be administered, for which the patient will have to drink oral contrast or receive rectal administration prior to CT. After the first postoperative week, CRP samples will be collected on demand, according to the consulting physician. If a major complication is diagnosed, immediate treatment will be commenced as soon as possible and as appropriate. All major complications are to be confirmed in reoperation or suspicious drain fluid upon percutaneous drainage and culture.

**Fig. 1:**
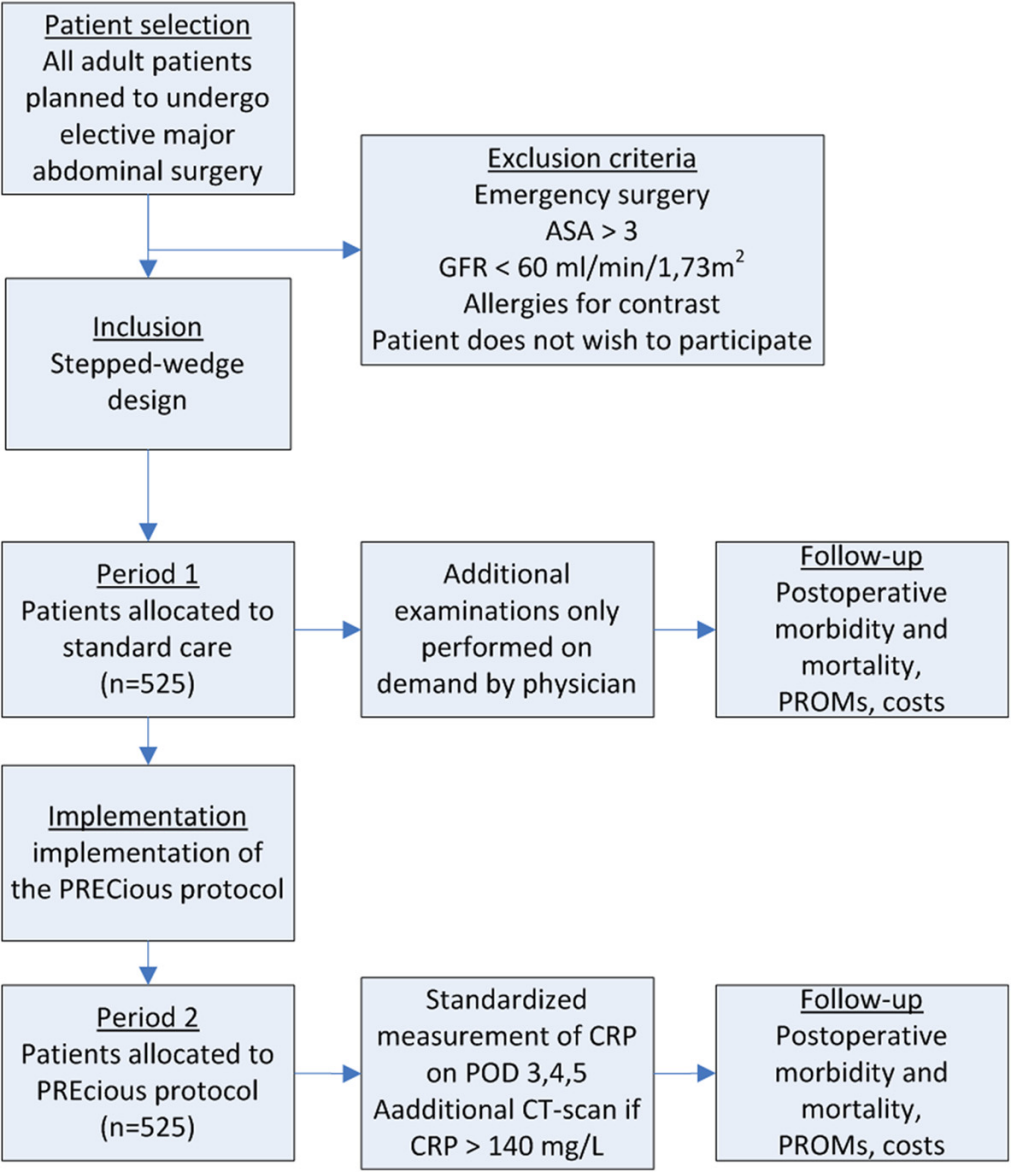
Flow chart for the stepped wedge design of the PRECious trial. ASA=Americal Society of Aneasthesiologist score, GFR=Glomerular filtration rate, PROMs=Patient Reported Outcome Measures, POD=Postoperative day

To allow for correct implementation of the protocol a transition period of month is deemed necessary before starting measurements in the intervention group. A flow-chart depicting the design of the study is displayed in Fig. 1.

#### Postoperative management

Besides differences in postoperative controls, postoperative management will be similar in both groups. Early mobilization will be encouraged, starting with sitting out of bed the first day postoperatively. A dietician will monitor intake. Patients will be discharged when they pass stools, are able to drink and eat sufficiently and are comfortable with oral analgesia. Follow up will be scheduled at the outpatient clinic 3 and 12 months after the index operation. Here PROMs will be collected and morbidity monitored.

## Discussion

In an era of advancements in surgical techniques and fast-track care, both modalities aiming to decrease morbidity and mortality after major abdominal surgery, there is currently no postoperative quality control algorithm available. A quality control algorithm should aim at safe discharge and early diagnosis and treatment of complications [10, 12]. Den Dulk et al. demonstrated a decrease in the time taken to diagnose anastomotic leakage, by implementing a leakage score. Although no significant difference in mortality rates was observed (39 % versus 24 %; *p* = 0.24), their research supports the use of a postoperative quality control algorithm, although the optimal algorithm is yet to be determined [6, 7].

CRP has shown promising in multiple observational studies, with an optimal cutoff in our observational cohort study of 399 patients of 140 mg/L on the third POD [8, 20, 23, 27–29, 31, 41]. Information on the predictive value of dynamic changes in CRP is not available, but will be monitored, comparing the levels on POD 2 to 5. POD 1 levels are not measured, because a peak is not observed until POD 2 [23].

The effect of standardized measurement of CRP levels on diagnosis of postoperative complications has yet to be determined. Here we propose a standardized postoperative quality control algorithm including repeated CRP measures and additional CT if CRP levels exceed 140 mg/L on the third, fourth and fifth POD [2]. Aiming for a decrease in morbidity and mortality associated with major complications after major abdominal surgery a prospective, randomized study is deemed necessary.

## Trial status

The scientific research committee approved the design of the PRECious-trial. Approval by the medical ethics committee of the VU University Medical Center, Amsterdam was obtained on 26 May 2015 (Protocol ID 2015.114 – NL43543.029.15). The trial is not yet recruiting.

## Additional file

Additional file 1:
**SPIRIT item checklist.** Checklist that addresses the separate items of the SPIRIT guidelines and where they are listed in the manuscript. (DOC 132 kb)

## Abbreviations

CRP: C-reactive protein
CT: computed tomography scan
EQ-5D: EuroQol 5D questionnaire
GIQLI: gastrointestinal quality of life index
IL-6: interleukin 6
MAS: major abdominal surgery
POD: postoperative day
PROM: patient reported outcome measure
SIRS: systemic inflammatory response syndrome
TNF-α: tumor necrosis factor α
WBC: white blood cell count
